# Work interruptions of office workers: The influence of the complexity of primary work tasks on the perception of interruptions

**DOI:** 10.3233/WOR-220684

**Published:** 2024-01-12

**Authors:** Vera B. Rick, Christopher Brandl, Alexander Mertens, Verena Nitsch

**Affiliations:** a Institute of Industrial Engineering and Ergonomics, RWTH Aachen University, Aachen, Germany; b Fraunhofer Institute for Communication, Information Processing and Ergonomics FKIE (Fraunhofer FKIE), Aachen, Germany

**Keywords:** Workload, digital work, office work, knowledge work, routine work, task complexity

## Abstract

**BACKGROUND:**

Research demonstrates that work interruptions are considered one of the most common work stressors. Understanding the mechanisms of work interruptions is therefore vital to reducing worker stress and maintaining performance.

**OBJECTIVE:**

The aim of this research is to investigate the influence of the frequency of work interruptions on subjective workload in the context of office work. Specifically, the mediating influence of interruption perception as well as the moderating influence of the complexity of the primary task are examined.

**METHOD:**

The work interruptions of 492 office workers in Germany were collected by means of a one-day diary study. A mediation model and a conditional indirect effect model were calculated to examine the influence of interruption frequency on subjective workload, mediated by the individual perception of these interruptions as well as moderated by the complexity of the primary work tasks.

**RESULTS:**

The analyses indicated a significant mediation and moderation. This implies that, on the one hand, the perception of work interruptions significantly mediates the relationship between the frequency of work interruptions and subjective workload. On the other hand, more complex primary work tasks seem to strengthen the positive relationship between interruption frequency and perceived interruption overload.

**CONCLUSION:**

The study underlines that work interruptions need to be considered in a much more differentiated way than is currently the case. Both in research and in terms of intervention measures in the work context, the various influencing factors need to be identified for an assessment of the effects on the working person to be possible.

## 1 Introduction

Work interruptions come in various forms, such as e-mails, instant messages, or colleagues looking for a conversation partner. Research demonstrates that work interruptions are considered one of the most common work stressors [1, 2]. Information workers spend on average more than two hours per day dealing with work interruptions [3] and often get caught up in “distraction chains” before resuming to their main tasks. These interruptions incur recovery costs (i.e., the time it takes to resume work after an email interruption), which typically amount to several minutes per interruption [4]. This makes it particularly necessary to investigate the challenges and especially the effects of work interruptions for information workers.

Work interruptions can be defined as a temporary interruption of goal-directed actions [5]. Although work interruptions can be externally or internally initiated, this research focuses on externally initiated interruptions caused by unplanned tasks related to the completion of a main task [6]. According to the action regulation theory, interruptions can disrupt the sequential action regulation process and thus be regarded as a regulation obstacle [7]. Thus, higher workload is to be expected, as the interrupted person must engage in a new task and resume to the former task later, i.e. a change of task is required, which necessitates mental regulation [5], shifting attention and adjusting the goal of action [8]. Resulting overload can lead to the individual’s disability to cope with the job demands, leading to slower work rates, including slower responses to critical events, as well as higher error rates, for example [9]. In the long-term, this increases the risk of serious health issues, long-term sick leave, or early retirement [10].

Looking at previous research on this topic, it is striking that only a limited number of studies have investigated work interruptions in the context of office work. Most of the research to date has been conducted in other occupational contexts (particularly healthcare) or in laboratory experiments [11, 12]. However, office workplaces represent a significant economic factor. In Germany, 71% of all employees work at least some of the time at an office workplace [13]. Office work refers to work at a desk workstation and is often accompanied by the intensive use of digital information and communication technologies. The work tasks of office work can be diverse, with predominantly routine-based requirements or, conversely, knowledge-based requirements, or a combination of both aspects. This makes it difficult to analyze the effects of work interruptions in these workplaces, as it can be assumed that these different main tasks can lead to different effects of interruptions [11, 12]. A review of previous literature shows in particular studies in the context of knowledge work [14–17] as well as among IT-professionals [18–21] and call-center employees [22], but a comparison of results is difficult due to the different study designs and variables collected. In addition, most studies lack an overview of work tasks, which prohibits more detailed deductions regarding the influence of work interruptions for office workers. However, taking into account laboratory findings, it may be assumed that the complexity of the interrupted task (i.e., the primary work task) serves as a key moderator, since interruptions of more complex tasks leads to greater information processing burden and higher mental effort [23, 24]. Highlighting this, previous research has even demonstrated that interruptions during routine-based work tasks can actually have positive effects, as they allow workers to engage in activities that are important for emotional well-being, job satisfaction, and continued productivity [25]. Moreover, it is shown, that in monotonous activities, interruptions which divert the attention can contribute to the activity’s variety and intellectual stimulations, thus serving as a source of work enrichment [26]. Accordingly, it must be expected that interruptions can have different effects even within office workplaces, depending on the primary work tasks.

With regard to the operationalization of work interruptions in field research, it appears that studies under this framework usually adopt a subjective approach, often focusing on frequency of interruptions and involving participants subjectively estimating the number of work interruptions (e.g., [6, 27]). However, research already shows that the individual evaluation of work demands in general seems to be an important mediating pathway between work demands and their effects on work attitudes and mental workload. Furthermore, it is confirmed, that there are certain organizational variables which influence individual appraisal of work interruptions and thus the effects of work interruptions for the working person [28]. An important distinction to physical stressors, is that psychosocial stressors are determined entirely, or at least in part, by the way people perceive them (e.g., require cognitive assessment) [29]. Regarding the individual evaluation of work interruptions, initial research has addressed the perception of work interruptions in the context of technostress research under the term interruption overload [14, 30]. Following these authors, interruption overload describes the extent to which individuals perceive that they receive more interruptions than they can effectively handle. Interruption overload originates in the cognitive resources needed when a person switches focus between tasks and is grounded in the literature on cognitive workload. It refers to the ability of people to achieve a given level of performance when limited mental resources are available [31]. It can be assumed that when a primary task is interrupted by an external stimulus, attention is consciously or unconsciously diverted from this primary task. At this point, a decision must be made whether to focus on the new task, divide attention between tasks, or ignore the interruption. Even if the decision is made not to pay attention to the interrupting stimulus, this decision is itself a decision point about whether or not it is worth paying attention to the stimulus, which may increase cognitive load.

In order to gain a more detailed insight into the influencing factors as well as effects of work interruptions at office workplaces, the present study conducted a one-day diary survey on work interruptions among office workers in order to analyze whether the complexity of the primary work task leads to different effects of work interruptions. Two hypotheses were formulated to be answered in the context of this research. The first hypothesis concerns the influence of perceived interruption overload as a mediating variable between the relationship of interruption frequency and subjective workload. Here, when operationalizing work interruptions, the objective number of these is included in the analysis rather than the subjectively estimated number. Continuing, it is assumed that professionals who are interrupted during more complex primary work tasks are more negatively affected by work interruptions. The second hypothesis therefore focuses on the moderating influence of the complexity of the primary work task:

**Hypothesis 1:** A higher frequency of work interruptions is significantly positively related to a higher subjective workload; this relationship is mediated by perceived interruption overload.

**Hypothesis 2:** A higher complexity of primary work tasks strengthens the positive relationship between frequency of work interruptions and subjective workload. 

## 2 Method

### 2.1 Procedure

The present study was conducted in the form of a one-day diary study in January 2022 to examine the association between the frequency of work interruptions and subjective workload of the working person. Data were collected via an online survey by participants which were recruited through a survey panel provider. The survey panel was accessed through *Bilendi GmbH*, a service provider who have access within their panel to registered natural persons who voluntarily participate in surveys. Contact with the participants in this study was therefore established by the panel provider through its standardized process of inviting potential participants. A random sample was drawn from the entire survey panel, provided they met the inclusion criteria of the survey. The inclusion criteria required that respondents were at least 18 years old, no older than 67 years (retirement age in Germany), worked exclusively at an office workstation, and were employed full-time (at least 35 working hours per week). Office work was operationalized with two items. Only participants who work predominantly at a desk workstation with a computer were included in this survey. Participation was voluntary, anonymity and confidentiality were guaranteed. Upon full participation, respondents received monetary compensation for their participation from the panel provider. As the study fulfilled a list of standard criteria (e.g. anonymized participation, adult participants, no intrusive measures, no deception), further ethical approval was waived.

In a first step, the subjects were informed about the content and procedure of the study as well as the data protection regulations. This information was provided to the participants in written form via the questionnaire platform. For potential questions, a designated contact person was given. In addition, the demographic data were verified regarding the inclusion criteria. In this first step, 985 participants were recruited who agreed to take part in the study. After giving their consent, subjects could choose any day within a two-week period on which they wanted to participate. On this day, the subjects received the first part of the questionnaire in the morning before their workday, following which all work interruptions during the workday were noted. The second part of the survey was filled out at the end of the workday with regard to perceived interruption overload and overall workload to be assessed. In this phase of the study, 615 participants took part in the study (response rate: 62.44%), of which 492 could be included in the analysis (49.95%) after the quality check of the data.

### 2.2 Sample

The sample consisted of 492 full-time office employees working in Germany. The sample included 45.5% female participants and 53.9% male employees, representative of the German working population. The age of participants ranged from 21 to 67 years with an average age of 43.9±11.9 years. The age grouping also took into account the representativeness of the German working population. The majority of participants were regular employees (65.6%), a further 27.0% were team leaders or middle managers, while 5.7% were senior managers. A complete sample description is provided in Table 1.

**Table 1:** Sample description

		**N**	%
Gender	Female	224	45.5
	Male	265	53.9
	Other	3	0.60
Age	18 –29	87	17.6
	30 –39	97	19.7
	40 –49	133	27.0
	50 –59	128	26.0
	60 –67	48	9.7
Position	Management	163	33.1
	Employee	321	65.2
	Other	8	1.6
Professional experience [very low (1) to very high (7)] (M/SD)		5.9 (1.1)
Working hours / week [hours] (M/SD)		40.1 (4.8)
Technological affinity [very low (4) to very high (1)] (M/SD)		1.6 (0.7)

### 2.3 Measures

After screening potential participants regarding inclusion criteria, the first part of this survey was to be completed in the morning before the start of the workday (pre-work measurements), the second part during the workday, and the third part at the end of the workday (post-work measurements). The pre-work and post-work measurements are described in more detail below; the measurements during the workday consisted of noting all interruptions during the workday in a previously distributed template.

#### 2.3.1 Screening and pre-work measures

The first part of the survey included a screening of potential participants to test for inclusion criteria. Demographic data of the participants were collected (gender, age, position, work experience, weekly working hours, type of workplace, and questions about work equipment). Participants who met the inclusion criteria received an additional questionnaire designed to elicit psychosocial requirements and resources, which, however, are not part of this analysis. In addition, the procedure of the study was explained in more detail, especially what a work interruption is and when and how participants should note them. This information was provided to the participants in written form via the questionnaire platform, for potential questions a designated contact person was given. An overview of the information collected that is relevant for this analysis is given in Table 2.

**Table 2:** Pre-work measures

Scale	Item		Response options
Information about the person	Please specify your gender		Female, Male, Other, I don’t want to say
	In which year were you born?		[open answer]
Information about the workplace	How much of your work activity do you perform at a desk workstation?		None at all (1) - (Almost) all (5)
	How many of your work tasks require you to work with a computer or laptop?		None at all (1) - (Almost) all (5)
	Please specify your average working hours per week		[open answer]
	What employment do you have in your company?		Upper management, Middle management, Team leader, Employee, Other
	How would you rate your professional experience?		Very low (1) - Very high (7)
Information about technologies used	How often do you use the following devices during work, for example to complete your work tasks but also to check mails or the appointment calendar?	Computer/Laptop/Tablet
		Smartphone	Never / Very rare (1) - Very often (5)
		Smartwatch	
Information on technology affinity	How would you rate your skills in using information and communication technologies (phone, email, smartphone, web video services, etc.)?	Very experienced and technically inclined (1),	
		Good at handling but not very technically inclined (2),	
		I can handle most communication techniques (3),	
		I find communication techniques very difficult to use most of the time (4)	

#### 2.3.2 Post-work measures

In the evening, the participants were asked to rate their perceived interruption overload, i.e., the extent to which they have received more interruptions than they can effectively process and manage using the corresponding scale [14]. Since there is currently no validated German version for this scale, the translation was done by the researchers of this work. In the translation process, three independent translation drafts were created, from which the final version was developed in joint consultation. The scale was rated on a 5-point Likert scale with values ranging between “*Not at all*” (1) to “*Fully agree*” (5), whereby a higher value describes a higher perceived interruption overload. Furthermore, the complexity of today's work tasks was assessed using two items of the Work Design Questionnaire (WDQ), whereby an already validated German translation was used [32]. Response options ranged from “*Not at all*” (1) to “*Fully agree*” (5), with a higher value describing more complex work tasks. In addition, participants were asked about their subjective workload during the workday using the Raw TLX scale, which is based on NASA TLX scale, using the six subscales: Mental, physical, and temporal demands, frustration, effort, and performance, without pairwise comparisons [33]. The total workload is calculated using the mean value of the subscales; values range between *low* (0) and *high* (100) total workload (Table 3).

**Table 3:** Post-work measures

Scale	Item		Response options
Raw TLX [33]	Please rate your workday today based on the following questions:	How much mental and perceptual activity was required today? Were your tasks easy or demanding, simple or complex?	Very low (0) –Very high (100)
		How much physical activity was required today? Were your tasks easy or demanding, slack or strenuous?
		How much time pressure did you feel due to the pace at which the tasks or task elements occurred today? Was the pace slow or rapid?
		How successful were you in performing your tasks today? How satisfied were you with your performance?	Perfect (0) –Failure (100)
		How hard did you have to work (mentally and physically) to accomplish your level of performance today?	Very low (0) –Very high (100)
		How irritated, stressed, and annoyed versus content, relaxed, and complacent did you feel during the tasks today?
Task complexity [32]	How would you rate your work tasks today?	My tasks today were simple and straightforward. (inverted)	Not at all (1) –Fully agree (5)
		My tasks today required a lot of thinking.
Frequency of work interruptions –counted during one full working day by the participants	How often were you interrupted by others today? (“Others” refers to all persons with whom you had contact during today’s workday and which caused an interruption of work for you. This does not have to be a person, but can also be an email, a phone call or a text message)	Email Instant messages Phone calls Virtual meeting Face-to-face contacts	[open answer]
Interruption overload [14]	How do you rate the messages and meetings received today? During my work day...	... I felt pressured by interruptions.	Not at all (1) –Fully agree (5)
		... I felt rushed because I was constantly interrupted.
		... I felt overloaded because I had to deal with more interruptions than I could handle.
		... I felt burdened because I had to deal with many interruptions.
		... I felt pressured by interruptions.

### 2.4 Analyses

First, a multi-stage screening of the responses received was carried out to ensure sufficient data quality. Participants with implausible completion times were excluded, using the relative speed index with a lenient cut-off of 2.0 as criterion [34]. In addition, two attention check items were included, which participants had to pass [35]. Finally, the counted work interruptions were checked for outliers using boxplot diagrams. Values that were more than 2.5 times the interquartile range away from the third quartile were checked for further use in the analysis by checking all data for plausibility, for example with regard to the stated occupation and the open answers given to describe the interruptions.

**Fig. 1:**
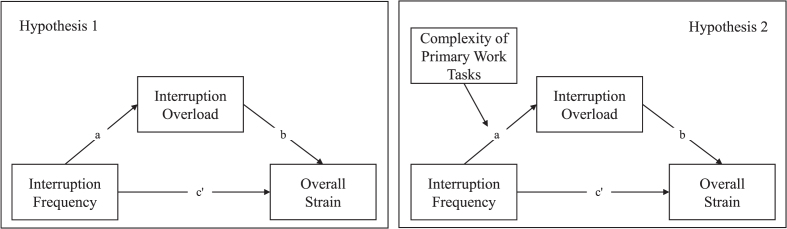
Analyzed mediation model, answering hypothesis 1 (left figure) and conditional indirect effect model, answering hypothesis 2 (right figure).

In order to ensure that a possible existing effect can be found with sufficient probability, the necessary sample size was determined in advance. For this purpose, the simulation-based calculation was chosen, since it takes into account that both a-path and b-path have to be interpreted. A power of at least 0.80 was assumed, and it was taken into account that percentile bootstrapping will be used. Expecting a medium effect size of a-path and b-path a sample size of N = 78 is necessary, whereas expecting a small sample size for both paths a sample size of N = 558 is necessary [36] (for an explanation of a-path and b-path, see Fig. 1). For this study, the number of subjects was targeted at N = 558 participants. However, due to dropouts and the multi-stage screening procedure to ensure the quality of the data, the final sample size of N = 492 unfortunately fell just short of this target.

For hypothesis 1, a mediation model was calculated. Mediation or an indirect effect is when a mediator (M) transmits the causal effect of a predictor (X) on a criterion (Y). Mediation is moderated when the indirect effect of X on Y through one or more mediators (M) depends on a moderator (W), which on the one hand can be called moderated mediation or, following more recent research, conditional indirect effect [37, 38]. Such a conditional indirect effect model was calculated to answer hypothesis 2 (Fig. 1). Both models were analyzed using PROCESS procedure for R version 4 [38], which uses ordinary least squares regression, yielding unstandardized coefficients for all effects. In order to calculate an even more robust model independent of possible violations of normality and heteroscedasticity, bootstrapping with 5000 samples together with heteroscedasticity consistent standard errors (HC3; [39]) were employed to compute the confidence intervals. For a more accurate result, effects were deemed significant when the confidence interval did not include zero. The prerequisites for calculating mediation and conditional indirect effect models were checked, whereby the assumption of linearity was established and confirmed by visual inspection of the scatter plots after LOESS smoothing.

To assess the mediation effect, first the indirect relationship between frequency of work interruptions and subjective workload through perceived interruption overload was calculated, along with the confidence interval (CI), by using the PROCESS Model 4. This first step includes an assessment of the signs and significance levels of the direct paths between frequency of work interruptions and perceived interruption overload and frequency of work interruptions and subjective workload. In line with the proposed theoretical framework, the PROCESS Model 7 was used, to estimate the moderating effect of primary work task on the path between frequency of work interruptions and perceived interruption overload. To test for the presence of moderated mediation, the effect sizes of conditional relationships were compared when the moderator is one standard deviation below its mean (–1 SD), at its mean (M) and one SD above its mean (+1 SD).

## 3 Results

### 3.1 Descriptive statistics and correlation analyses

The participants stated that they were interrupted 25±29.42 times during their workday, of which 12±15.94 interruptions were due to emails, and around 7±10.57 interruptions due to phones calls. The remaining interruptions were due to instant messages, meetings and personal contacts (Table 4).

**Table 4:** Number of work interruptions per interruption type; N = 492

Work interruptions	M	SD	Min	Max
All interruptions	25.52	29.42	0	344
Emails	12.01	15.94	0	150
Phone calls	6.63	10.57	0	98
Instant messages	4.16	8.43	0	100
Meetings	0.84	1.36	0	8
Face-to-face contacts	1.88	11.86	0	256

The subjects rated their perceived interruption overload during the past working day with M = 2.10±0.99. Internal consistency was checked to verify the translation. Cronbach’s alpha was 0.94 for this scale, illustrating very good internal consistency. The complexity of primary work tasks was rated with M = 3.44±0.10 (items were to be answered on a scale from not at all (1) to fully agree (5)) and subjective workload was rated with M = 48.15±15.06 (items were to be answered on a scale from low (0) to high (100)).

The correlation analysis shows that the age of the participants was significantly negatively correlated with perceived interruption overload and furthermore significantly positively correlated with subjective workload. The complexity of primary work tasks, on the other hand, is significantly positively correlated with frequency of work interruptions, perceived interruption overload and subjective workload. The frequency of work interruptions is significantly positively correlated with perceived interruption overload and further significantly positively correlated with subjective workload. A detailed overview is given in Table 5. According to the results, age is included as a covariate in the further analyses.

**Table 5:** Correlation analyses

	Pearson correlation coefficient				
	1	2	3	4	5
Age (1)	1	0.142**	0.025	–0.103*	0.135**
Complexity of primary work tasks (2)		1	0.142**	0.347**	0.576**
Interruptions frequency (3)			1	0.393**	0.335**
Perceived interruption overload (4)				1	0.470**
Subjective workload (5)					1

*Note*. ** –*p* < 0.01, * –*p* < 0.05.

To check whether gender also has to be included as a covariate in the following models, t-tests for independent samples are calculated for all relevant variables. Based on the sample distribution, only female and male participants are compared. There was no statistically significant difference between female and male participants with regard to interruption frequency (t(487) = 0.808, *p* = 0.419), perceived interruption overload (t(487) = 0.169, *p* = 0.866), complexity of primary work tasks (t(487) = –1.841, *p* = 0.06) and subjective workload (t(487) = –0.357, *p* = 0.177). According to the results, gender of the participants is not included in the further analyses.

### 3.2 Mediating influence of the perception of work interruptions as overload

A mediation model was calculated to analyze whether frequency of work interruptions predicts subjective workload and whether the direct path would be mediated by the perception of work interruptions as overload. Bases on the analysis above, age is included as a covariate into the model. An effect of frequency of work interruptions on subjective workload was observed (β= 0.164, *p* < 0.001). After entering the mediator into the model, frequency of work interruptions predicted perceived interruption overload significantly (β= 0.397, *p* < 0.001), which in turn predicted subjective workload significantly (β= 0.424, *p* < 0.001). The relationship between frequency of work interruptions on subjective workload is partially mediated by the perceived interruption overload, indirect effect ab = 0.168, 95% -CI [0.126, 0.216] (Table 6).

**Table 6:** Conditional direct effect frequency of work interruptions on perceived interruption overload

	a-path			b/c'-path		
	β	SE	p	β	SE	p
Interruption frequency	0.397	0.001	<0.001		
Interruption overload				0.424	0.645	<0.001
Subjective workload				0.164	0.021	<0.001
Age	–0.113	0.0034	<0.001	0.175	0.049	<0.001

*Note*. N = 492. Model for the a-path R^2^ = 0.168, F(2, 489) = 49.199, *p* < 0.001. Model for b-path and c’-path R^2^ = 0.278, F(3, 488) = 62.663, *p* < 0.001.

### 3.3 Moderating influence of the complexity of primary work tasks

A conditional indirect effect model was performed to analyze whether frequency of work interruptions predicts subjective workload and whether the direct path would be mediated by perceived interruption overload and further, whether the complexity of primary work tasks moderates the relationship between interruption frequency and perceived interruption overload. Bases on the analysis above, age is included as a covariate into the model. The results show a positive, significant effect of the interaction term (frequency of interruptions and complexity of work tasks) (*b* = 0.003, *p* = 0.01), with ΔR^2^ = 0.007. Taking the conditional effects of the focal predictor into account, the analysis reveals that the relationship between frequency of work interruptions and perceived interruption overload is stronger when primary work tasks are more complex.

**Fig. 2:**
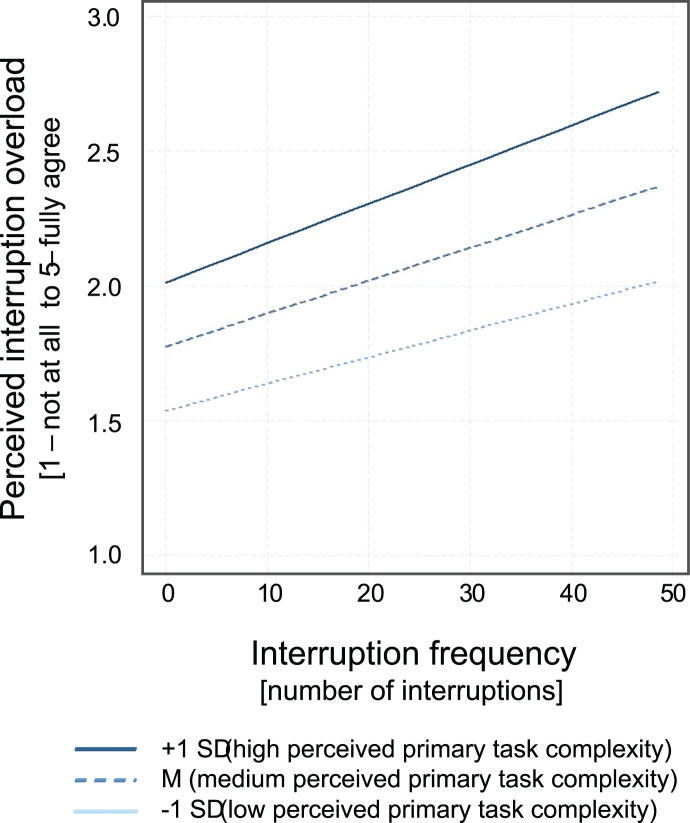
Moderation analyses with complexity of primary work tasks as a moderator.

Figure 2 illustrates the influence of the moderator, whereby the moderator is shown at the mean value (M) and at both one standard deviation below and above the mean value (±1 SD) of primary task complexity. Even with only a few work interruptions, a clear difference in perceived interruption overload between less complex (–1 SD) and more complex (+1 SD) primary tasks can be seen. This difference becomes even more obvious with many interruptions. Thus, the moderator strengthens the positive relationship between interruption frequency and perceived interruption overload.

The formal test of moderated mediation, which assesses the index of moderated mediation and the corresponding confidence intervals, did not confirm a significant analysis. A conditional indirect effect can therefore not be proven (Table 7).

**Table 7:** Conditional indirect effect

	Effect size	SE	LLCI	ULCI
-1 SD	0.009	0.001	0.006	0.012
M	0.012	0.001	0.009	0.016
+1 SD	0.115	0.002	0.010	0.020
Index of moderated	0.018	0.008	–0.001	0.034
mediation			

*Note*. N = 492; LLCI = lower limit confidence interval; ULCI = upper limit confidence interval.

## 4 Discussion

Focusing on office workplaces, the present study aimed to answer the question of whether perceived interruption overload serves as a mediator between work interruption frequency and subjective workload and, further, whether the direct relationship between interruption frequency and perceived interruption overload is moderated by the complexity of the primary work tasks. Results indicate a significant partial mediation of perceived interruption overload and a significant moderation of work task complexity, however, no conditional indirect effect was measurable. The findings are now discussed in more detail below.

First, the mediation model is discussed. Following the authors [14, 30], perceived interruption overload was examined as a mediator, whereupon partial mediation is shown. Partial mediation results from the fact that the direct relationship between interruption frequency and overall strain remains significant despite the addition of the mediator. Thus, perceived interruption overload, i.e., individual evaluation of work interruptions, has a significant and strong influence on subjective workload. The first hypothesis can therefore be confirmed. It can be inferred that the individual evaluation of work interruptions has a crucial importance for the overall subjective strain. This result can be considered decisive when it comes to identifying interventions and measures for long-term healthy working. It is only conditionally a matter of eliminating interruptions in the work context, but rather of identifying the factors that make the perception of work interruptions particularly serious. In other words, it is about finding out what factors and characteristics of a work interruption cause employees to perceive it as overwhelming. Previous studies have been able to provide some evidence of such factors and characteristics, but it is very clear that mainly experimental studies have addressed this research topic [11, 12]. These have manipulated the modality of interruptions, the complexity and similarity of tasks, the timing of interruptions, and the resumption delay (e.g., [40–42]), but in quantitative field research, a rather unidimensional view of interruptions can be observed, as mostly the frequency of work interruptions was queried. However, with regard to the results of the experimental research, a number of other characteristics can be considered significant, but they still need additional validation in field research. The goal should be to use these results to identify factors and characteristics on the one hand, and to develop appropriate interventions and health-related measures in dealing with incoming interruptions on the other. Previous intervention studies have focused primarily on reducing work interruptions, but not on how to deal with interruptions or what an interruption itself should look like. In addition to focusing on just reducing interruptions, the focus of previous studies has also been very much on health occupations. A systematic literature review shows that of 36 identified intervention studies, 35 were conducted in healthcare settings and primarily in hospital workplaces; of these, 20 studies focused exclusively on interruption reduction. These findings are, however, not transferable to the context of the office workplace [11].

In summary, there is some evidence of successful interventions to reduce interruptions in medical and nursing work. The research field still has potential for be expanded, on the one hand to offer insight to how interruptions are perceived in the first place, and on the other hand to determine what an interruption might look like to cause less overload. Further research in the context of office work must also be validated, since the results to intervention measures in the healthcare sector are most likely not be directly applicable.

In the second part of the analysis, a conditional indirect effect model was calculated, which included the complexity of primary work tasks as a moderating variable in the mediation model. The results show that the complexity of primary work tasks strengthens the positive relationship between interruption frequency and perceived interruption overload. Accordingly, a higher complexity of primary work tasks leads to a higher perceived interruption overload for the same interruption frequency. Therefore, the second hypothesis can be confirmed, even though it must be mentioned that the effect size is very small and no conditional indirect effect is measurable (i.e., there is no effect of the complexity of the primary work tasks as a moderating a-path variable on the overall strain on the working person). It should be noted that an existing small effect could not be found with sufficient probability due to the sample size, as the required sample size was not met. With a power of at least 0.80 and percentile bootstrapping, a sample size of N = 558 would be required to test for a small effect [36]. However, due to dropouts and the multistage screening procedure to ensure data quality, this goal was unfortunately missed with a final sample size of N = 492, meaning that small effects may not be detectable. However, the results indicate that interruptions are perceived differently, depending on characteristics of the interrupted task. The characteristics of the interrupted task form a group of previously studied moderators, with the complexity of the interrupted task being a key moderator. The findings are consistent which suggest that work interruptions in more complex primary tasks have a more negative effect on the interrupted person [23, 24]. The previous studies on this branch of research can therefore be substantiated in the context of office work and even extended to the fact that it is not only about the primary task actually interrupted at that moment, but it is generally about the type of tasks that a person works on in the course of the working day. The results are particularly interesting when considering possible positive effects of interruptions. Following the authors, interruptions during routine work may actually have positive effects because they allow them to engage in activities that are important for emotional well-being, job satisfaction, and continued productivity [25]. Moreover, it is shown that distractions of attention caused by work interruptions provide variety and intellectual stimulation in monotonous activities and thus serve as a source of work enrichment [26]. These results suggest positive effects that were not investigated in the present study but could bring further clarity to the understanding of work interruptions and their handling in the context of office work in the future.

### 4.1 Limitations

Due to the design of the study, there are some limitations that should be taken into account when interpreting the results. One limitation is that this study only examined one occupational context and the perspective of one country; transferability of the results to other countries and other occupational groups is limited and requires further research. Indeed, as the results show, effects already differ within this one occupational group, which means that transferability to other occupational groups can only occur within similar work tasks. Furthermore, due to the fact that only one day was considered for measuring work interruptions, longitudinal effects and causal inferences between work interruptions and the (negative) outcomes are not possible. Future studies attempting to model this causal chain should therefore use more advanced methodology, such as prospective designs and diary studies over a longer period of time. In addition, the method used required that respondents independently count their work interruptions, which can lead to errors, such as overlooking or increasing the number of work interruptions counted. In addition, focusing on work interruptions may cause them to be perceived differently than they normally would be if attention were not drawn to them. Moreover, the survey itself could be considered a work interruption, which is why the questionnaire was kept as short as possible so that the reported effects are unlikely to be caused by the extra effort associated with participation.

## 5 Conclusion

In today’s dynamic workplaces, work interruptions are common and unavoidable, exacerbated by changes in the world of work. This study has highlighted the importance of individual evaluation of work interruptions. It must be assumed that work interruptions do not always have the same effects and may have different consequences depending on the person interrupted and the task interrupted, amongst others. It is therefore crucial to consider work interruptions not only in terms of the possibility of reducing them, but also in terms of their nature and characteristics. In summary, the findings underline the importance of not focusing on a “reductionist” approach to work interruptions; rather, organizations need to focus on better managing interruptions in the workplace. However, this requires a better understanding of the effects of interruptions on employees and the circumstances in which they occur.

## Ethical considerations

Participation was anonymous, all participants were adults, no intrusive measures were used, and no deception of participants was used. A detailed explanation of the procedure is given in the methods section.

## Informed consent

The data has been collected anonymously. The participants have consented to the use of the anonymized data for scientific purposes. A detailed explanation of the procedure is given in the methods section.

## Reporting guidelines

Reporting is in accordance with the STROBE Statement for cross-sectional studies listed in the EQUATOR Network for Reporting Guidelines.
